# TYGS and LPSN in 2025: a Global Core Biodata Resource for genome-based classification and nomenclature of prokaryotes within DSMZ Digital Diversity

**DOI:** 10.1093/nar/gkaf1110

**Published:** 2025-10-29

**Authors:** Heike M Freese, Jan P Meier-Kolthoff, Joaquim Sardà Carbasse, Ayorinde O Afolayan, Markus Göker

**Affiliations:** Leibniz Institute DSMZ – German Collection of Microorganisms and Cell Cultures, Department of Bioinformatics and Databases, Inhoffenstrasse 7B, 38124 Braunschweig, Germany; Leibniz Institute DSMZ – German Collection of Microorganisms and Cell Cultures, Department of Bioinformatics and Databases, Inhoffenstrasse 7B, 38124 Braunschweig, Germany; Faculty of Applied Computer Science and Medical Faculty, University of Augsburg, Alter Postweg 101, 86159 Augsburg, Germany; Augsburg Bioinformatics Core Facility, University of Augsburg, Alter Postweg 101, 86159 Augsburg, Germany; Leibniz Institute DSMZ – German Collection of Microorganisms and Cell Cultures, Department of Bioinformatics and Databases, Inhoffenstrasse 7B, 38124 Braunschweig, Germany; Leibniz Institute DSMZ – German Collection of Microorganisms and Cell Cultures, Department of Bioinformatics and Databases, Inhoffenstrasse 7B, 38124 Braunschweig, Germany; Leibniz Institute DSMZ – German Collection of Microorganisms and Cell Cultures, Department of Bioinformatics and Databases, Inhoffenstrasse 7B, 38124 Braunschweig, Germany

## Abstract

The List of Prokaryotic names with Standing in Nomenclature (LPSN, https://lpsn.dsmz.de/) is an authoritative, expert-curated resource on prokaryotic nomenclature. Its sister database, the Type (Strain) Genome Server (TYGS, https://tygs.dsmz.de/), is a high-throughput platform for genome-based taxonomy. Here we present updates to these two platforms. New tools include improved interoperability with other DSMZ Digital Diversity databases, as well as easy-to-use query and access functions, and more comprehensive submission forms. The database content has expanded considerably, particularly through the inclusion of thousands of cyanobacterial names and the compilation of the List of Recommended Names for bacteria of medical importance (LoRN). LPSN now contains over 59 000 taxon names, and over 23 500 genome sequences have been added to TYGS. LPSN and TYGS have been updated to incorporate and reflect changes to the official rules governing prokaryotic nomenclature, as ratified by the International Committee on Systematics of Prokaryotes (ICSP) in recent years.

## Graphical Abstract

**Graphical Abstract:**
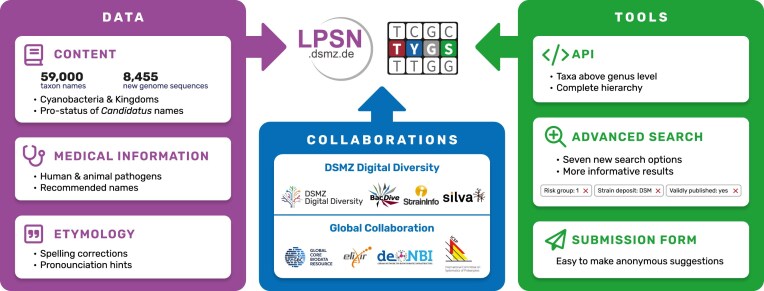

## Introduction

Due to the rapid changes in prokaryotic nomenclature, particularly the constant influx of new names [1–12], expert-curated database systems with semi-automated data collection are required to keep up with the literature. The List of Prokaryotic names with Standing in Nomenclature (LPSN) [13–17] provides comprehensive information on prokaryotic nomenclature and related data, and is updated weekly.

LPSN was founded over two decades ago by a single microbiologist, Jean Paul Euzéby, as a manually curated database [13–15]. Earlier, Prokaryotic Nomenclature Up-to-date (PNU) was established in 1993 as a service of the Leibniz Institute DSMZ—German Collection of Microorganisms and Cell Cultures. In 2020, after moving to DSMZ and integrating the PNU data, LPSN was relaunched based on a dedicated database infrastructure, including automated data import routines, a database-driven user-friendly website, an application programming interface (API), and a wealth of content-related additions [16]. Pages for individual taxon names were equipped with information on etymology, type-strain deposits, 16S ribosomal RNA (rRNA) gene sequences in FASTA format, International Nucleotide Sequence Database Collaboration (INSDC) 16S rRNA gene accession numbers, taxonomic and nomenclatural status, and many notes [16].

In December 2023, LPSN was selected by the Global Biodata Coalition as a Global Core Biodata Resource (GCBR). LPSN and TYGS are also registered as services of de.NBI (German Network for Bioinformatics Infrastructure), which represents the German Node within the ELIXIR network. LPSN is supported by the International Committee on Systematics of Prokaryotes (ICSP) [18, 19], the committee officially responsible for the regulation of prokaryotic nomenclature, the International Code of Nomenclature of Prokaryotes (ICNP) [20]. LPSN implements the ICNP comprehensively and transparently. The ICNP covers all prokaryotes, whether cultivated or not [20]. The ICNP remains the primary and most widely adopted framework for standardized naming of *Archaea* and *Bacteria*, and adherence to it is strongly recommended [21, 22].

In the 21st century, taxonomic classification has been greatly impacted by genome-scale data and associated techniques [23–29]. The ever-increasing growth of genome data [30] demands technical solutions that can efficiently and reproducibly standardize taxonomic analyses. Comparisons with the genome sequences of type strains are essential for the classification of novel strains [31]. Therefore, a reliable mapping between type strains, genome sequences, and taxon names must be established.

To address these requirements, the Type (Strain) Genome Server (TYGS) was introduced as a large database of curated type-strain genomes, combined with selected metadata and facilities for conducting genome-based taxonomic analyses at scale [17, 32]. Users can query the TYGS database using one or more uploaded genome sequences. Results include genome-scale phylogenies [33] and state-of-the-art estimates of species and subspecies boundaries for user-provided and automatically determined closest type genome sequences [17, 32, 34]. The metadata provided include nomenclature, synonymy, and associated taxonomic literature to facilitate taxonomic exploration [17, 32].

Accurate intergenomic Genome BLAST Distance Phylogeny distances [35] and digital DNA:DNA hybridization (dDDH) values are calculated and stored in the TYGS database. Most pairwise calculations among type genomes are precomputed, thus considerably accelerating the processing of these requests; distances involving user uploads are computed on demand. These distances are used for phylogenetic inference and also form the basis of the dDDH method, which is one of the most widely used indices of overall genome-relatedness (OGRI) [36] for *in silico* species delineation [35, 37]. This approach outperformed alternative OGRI indices [35, 37], which may yield inconsistent results due to a lack of standardization [38]. The need for an expert-curated nomenclatural database and for a database of verified type-strain genomes, their metadata and subsequent facilities for taxonomic analysis has been highlighted previously [17]. Access to a broader selection of “reference genomes”, including nontype strains, may lead to taxonomic misinterpretations, as they may be confused or misinterpreted as representing the nomenclatural types.

The establishment of LPSN at the DSMZ enabled the two databases to become strongly interconnected [16, 17, 32], further increasing their usability. TYGS and LPSN became a pair of synergistic, database-driven platforms that cover the core fields of prokaryotic (genome-based) taxonomy. They also provide guidance, which is especially useful for nonexperts, in a field where it can be difficult to choose between a plethora of tools and data entries. In this study, we present an update on TYGS and LPSN, including improvements and additions made since the last publication [17].

## TYGS and LPSN within DSMZ digital diversity

In 2023, LPSN and TYGS became part of the Digital Diversity Consortium newly established at DSMZ (https://hub.dsmz.de). The consortium encompasses the development of nine life-science databases, four of which are Global Core Biodata Resources. It facilitates the further development of the cross-connections between TYGS and LPSN and other databases, including SILVA [39], Bac*Dive* [40], and the relaunched StrainInfo (https://straininfo.dsmz.de). A federated search has been developed across all Digital Diversity databases, including LPSN. With the support of de.NBI, LPSN, and TYGS organize workshops to educate participants on how to use the resources and gather feedback to improve future development.

Various cross-connections between the databases have already been established, building on those previously reported [17] linking from TYGS and LPSN to Bac*Dive* [40]. For instance, the DSMZ catalogue, SILVA [39], Bac*Dive* [40], and StrainInfo now link to the relevant LPSN taxon pages. The LPSN API has been expanded to facilitate the import of data by other databases and the creation of links.

These expansions have enabled the creation of an LPSN-centred instance of TaxMap (https://taxmap.dsmz.de/) by the SILVA team [41]. TaxMap is an adaptation of EukMap, which was originally developed by the SILVA team as part of the UniEuk project [42], which provides a taxonomic framework for protistology. Several changes were made to the EukMap application to create an alternative tool for browsing the LPSN taxonomic hierarchy. Nucleotide sequence accession numbers from Bac*Dive* [40] have been added, primarily covering environmental sequences and those not derived from type strains.

## New data and new tools

**Figure 1.**
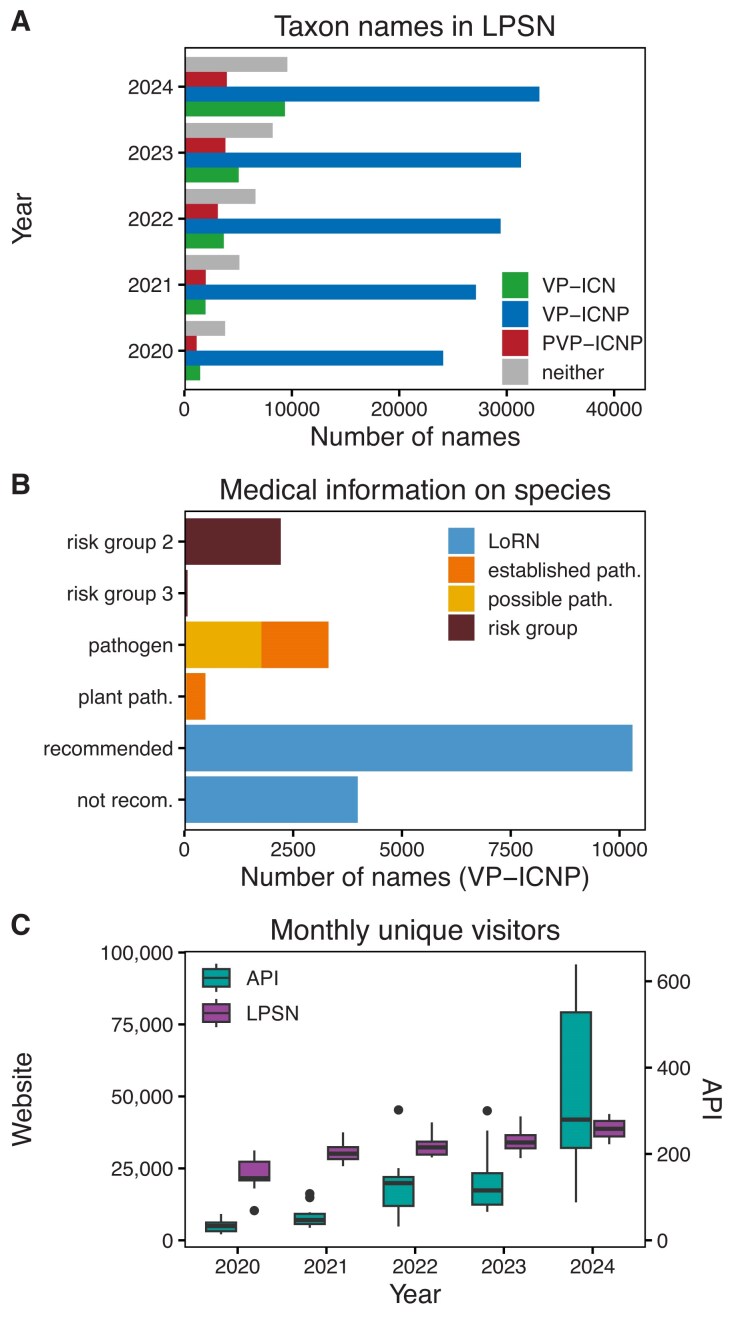
(**A**) Cumulative number of prokaryotic names included in LPSN grouped by completed year and nomenclatural status (VP-ICNP, validly published under the ICNP; VP-ICN, validly published under the botanical code; PVP-ICNP, pro-validly published under the ICNP; neither, neither validly published nor pro-validly published). (**B**) Current number of species names validly published under the ICNP classified as risk groups 2 or 3, recognized as possible or established pathogens (“path.”), and included in LoRN as either recommended name (correct name) or not recommended (“recom.”) name (synonym). (**C**) Median unique visitors per month to the LPSN website (left axis) and API (right axis) over the completed years. Box plots show the median, as well as the 25th and 75th percentiles for each year, while the whiskers show 1.5 times the interquartile range. Values outside this range are shown as outliers.

The scope and content of LPSN have been described in detail in previous publications [13–17]. Key components include the authority of a taxon name, reflecting its original publication and connecting nomenclature with scientific literature; synonymy of names; and nomenclatural types. While these components have not changed, the LPSN database has grown significantly. Figure 1 shows the number of taxon names in LPSN in each completed year since its relaunch. As previously mentioned [17], the total number of names that become validly published each year continues to increase. At the time of writing, LPSN contains more than 59K taxon names, more than 34K of which are validly published under the ICNP. In addition to its, on average, weekly updated adding new taxon names and associated information, LPSN has incorporated a substantial amount of supplementary data and introduced a variety of new features since the last publication in 2022 [17].

In collaboration with the *Ad Hoc* Committee on Mitigating Changes in Prokaryotic Nomenclature (CoMicProN) [43] of the ICSP, LPSN has compiled the List of Recommended Names for bacteria of medical importance (LoRN) [44, 45]. This list has been implemented directly within LPSN, combining a broad selection of bacteria of potential or established medical importance [46] with a careful selection of the correct name [45, 47]. The list is updated periodically. At the time of writing, more than 14K names have a LoRN tag in LPSN, 10K of which are recommended, i.e. are considered the correct name (Fig. 1). Other features have been included in conjunction with LoRN. The LPSN Advanced Search now allows filtering by LoRN, as do the CSV download and the API. The page of each taxon name included in LoRN now displays the relevant information as part of the taxonomic status and in the notes section. Earlier, more informative taxonomic status values were introduced, mainly for names that are not validly published.

The LPSN team has completed its screening of data on human, animal and plant pathogens, augmenting the pathogenicity information [46]. Added features on the taxon submission page include the ability to submit suggestions for notes on strains previously placed in other taxa, as well as suggestions for host relationships to be considered, all of which can be done anonymously. LPSN pages for individual taxon names now indicate whether a species was generated by splitting another species and display the number of PubMed records containing the respective name. LPSN has also imported risk group data [48] from additional countries.

A large number of cyanobacterial names [49] have been added to LPSN, following the ICSP’s acceptance [50] of names validly published under the botanical code [51] also under the ICNP [20]. At the time of writing, LPSN contains more than 9000 cyanobacterial names (Fig. 1). Additional checks have been introduced for these names, bringing the level of internal quality control closer to that for names validly published under the ICNP. During this process, information on numerous herbaria and algal culture collections has been added to the LPSN culture collections page. LPSN now provides links to culture collection catalogues for deposits that cannot be linked directly to a specific catalogue page. In these cases, the link is only placed on the acronym of the culture collection.

In 2024 the ICSP has ratified [52] a proposal to formally integrate *Candidatus* names into the ICNP [20], informally known as “Best of Both Worlds” [22]. *Candidatus* names can now have a nomenclatural pro-status, i.e. they can be pro-validly published, pro-legitimate, and pro-correct, in analogy to the nomenclatural status of non-*Candidatus* names. LPSN displays these pro-status values on individual taxon pages and makes them available via the advanced search function. At the time of writing, LPSN contained nearly 4000 pro-validly published names (Fig. 1). The LPSN statistics page was created to track the numerical development of LPSN using freely available figures. It now includes new graphics, such as the overall number of names validly published under the botanical code, validly or pro-validly published under the ICNP, or neither.

As noted earlier, many of the names of prokaryotes proposed in effective publications are not validated [53]. Expanding its scope to include names that are not validly published allowed LPSN to conduct monthly validation campaigns, informing authors of the need to validate the names they had proposed [17]. Such actions increase nomenclatural stability, which is reflected in LPSN through changes to the nomenclatural status of a name upon validation. These campaigns have now been augmented by the LPSN team’s own actions, such as traversing all names not published validly since 1980 [54], collecting missing information where possible, e.g. evidence of the deposit of the type strain in two culture collections in two different countries [20], and requesting the validation of names where possible [55].

In 2023 the domain and kingdom categories [56] have been incorporated into the ICNP [57], followed by proposals of two domain names and several kingdom names [58, 59]. The kingdom category is now fully included in the LPSN hierarchy browser. As part of this work, a larger number of synonyms have also become accessible via the taxonomic hierarchy. The LPSN API now delivers names above genus rank, provided they are validly published under the ICNP. It is possible to reconstruct the entire LPSN hierarchy using the API. In order to facilitate usage by commercial firms, the copyright licence for all LPSN data, including those available via the API, has been changed from CC BY-NC 4.0 to CC BY-SA 4.0.

The nomenclatural types of classes and subclasses have been changed from order to genus, reflecting the corresponding emendation of the ICNP [60]. A major overhaul of the LPSN entry page on nomenclature has been conducted. The nomenclature page now indicates which publications are official ICSP publications and which are proposals to amend the ICNP. It also shows which publications are revisions or approved amendments of the ICNP. The list of Judicial Opinions issued by the Judicial Commission of the ICSP has been kept up to date, including Opinions 128–132 [61–65]. The advanced search function allows users to filter by nomenclatural status and “proposed as” entries on each LPSN taxon page.

Following the 2022 revision of the ICNP [20], spelling corrections to prokaryotic names can be made more freely. LPSN now provides a better alternative for many incorrectly formed epithets. On the LPSN taxon submission page, users can submit etymological suggestions for entries that are missing, considered incomplete, or otherwise inadequate. Prof. Aharon Oren has provided literally thousands of cyanobacterial etymologies. LPSN has already checked many syllabifications, including those for all (pro-)validly published taxon names of genus rank or above. For names with checked syllabifications, hints on how to pronounce them are provided [66]. The LPSN glossary has been expanded accordingly. In the case of adjectives and participles that are identical in some of the three grammatical genders, the gender of the genus name is now enforced in the etymology. The combining form (stem) is shown for each genus name, along with its gender.

**Figure 2.**
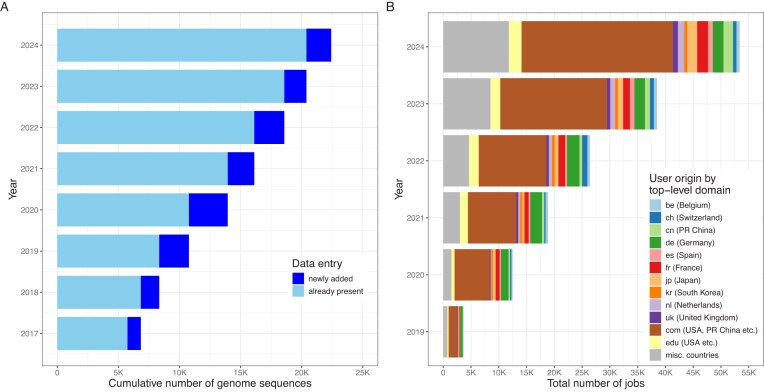
(**A**) Statistics on the cumulative growth of TYGS genome sequences of a given type (strain) per month for all completed years. (**B**) Noncumulative number of TYGS job submissions per month for all completed years since its establishment in 2019. Job submissions are grouped according to the top-level domain of the e-mail address included in each submission. Wherever possible, the e-mail domain was matched to the country of origin.

The nomenclatural type is the entity with which a taxon name is permanently associated [20]. For prokaryotic species and subspecies with a validly published name, the nomenclatural type is the type strain. Cultures of type strains therefore firmly link taxon names to genome sequences (when genome sequences are available). For pro-validly published *Candidatus* names, the nomenclatural type can be a genome sequence and the connection to the TYGS database is direct. At the time of writing, the TYGS database comprised more than 23K type(-strain) genomes (Fig. 2), more than 8K of which were added in the last 2 years, reflecting the increase in the number of species and subspecies names collected by LPSN. This increase is caused by the broadening of LPSN’s scope, as well as by the increased number of names proposed per year and possibly also by increased efforts at sequencing type-strain genomes.

Despite requiring greater computational power to handle the increased volume of data, the default TYGS upload limit has been increased from 20 to 50 genome sequences. Throughput and scalability have been improved by optimizing user job selection and scheduling queries further. Specifically, jobs are now scheduled according to a fair-share policy, whereby tasks with a relatively small computational footprint are prioritized over more resource-intensive ones and older submissions are favoured over more recent ones.

The Genome-to-Genome Distance Calculator (GGDC) predates the TYGS and remains a popular online tool for calculating dDDH values for *in silico* (sub-)species delineation [35, 37]. Users do not need to submit genome sequences to either TYGS or GGDC. The systems also accept various kinds of INSDC identifier [17, 32, 35, 37]. Mapping between these types of identifier (e.g. between an assembly accession number and a nucleotide sequence accession number) for sequence download has now been made more efficient by using local identifier databases.

The final step of the comprehensive genome-based taxonomic analyses available in the TYGS involves genome-scale and proteome-scale phylogenies, 16S rRNA gene trees and type-based clusterings at the species and subspecies levels. The way in which users are presented with these results has also been improved. Design revisions were made to add sections, in-page navigation and clearer warnings or input validation feedback. Previously, the results of extended 16S rRNA gene phylogenetic analyses were only available via e-mail through the GGDC phylogeny server [17]. These analyses are taxonomically relevant, as there are still validly published taxon names lacking a type genome sequence. To further streamline interaction with various TYGS results, the extended 16S rRNA gene analysis has been integrated into the TYGS results page.

GGDC analysis outcomes are now also displayed on TYGS result pages, which makes them easier to interpret. Although the results of TYGS and GGDC can be found on temporarily available websites, it is important to inform users once a calculation has been completed. TYGS emails now include identifiers that make it easier to organize TYGS jobs and results, especially when users submit several jobs simultaneously. The accompanying TYGS web service saw major additions, including new analysis and visualization options (e.g. the ability to download digital DNA–DNA hybridization results as TSV files and a ‘quick summary’ button on each result page).

## Usage and outlook

LPSN (Fig. 1) and TYGS (Fig. 2) are highly recognized and serve microbiologists and others worldwide, which is reflected in their user statistics. In the case of LPSN, these statistics are based on accesses from unique IP addresses to the LPSN website or API, collected via Matomo (https://matomo.org/). In the case of TYGS, however, the number of submitted jobs can be counted. As of 8 January 2024, TYGS had already delivered a total of 100 000 jobs. Interest in both systems continued to increase, with many new users joining every month. At the time of writing, more than 20 000 different users worldwide had used TYGS (Fig. 2) to conduct more than 180 000 analyses involving more than 96 million genome comparisons. The unique visitors of LPSN increased over time up to a yearly median of 38 700 monthly users in 2024 (Fig. 1), these users originated from 221 different countries in 2024. Especially, data retrieved via the API increased strongly (Fig. 1), which will be considered for future developments.

Based on contemporary taxonomic activities and the exploration of additional literature sources, the growth of TYGS and LPSN is expected to continue in the coming years. This includes uncharted publications from the past, particularly from before 1980 [54], further improved routines for finding contemporary sources and taxon names within these sources, and the activities of the ICSP, particularly with regard to the nomenclatural types of *Candidatus* names [22, 52]. TYGS will also benefit from these developments. It may be augmented with more comprehensive phylogenomic trees and used more frequently to inform the LPSN taxonomic hierarchy. The List of Recommended Names for bacteria of medical importance [44, 45] could be extended to encompass phytopathogenic bacteria [67–69]. The LPSN API may be expanded further with additional entries or features, and LPSN may assign digital object identifiers for taxon names. Data available via the LPSN API may be converted into an ontology of ICNP terms and taxon names and linked to ontologies developed in other databases. In this way, and in other ways, TYGS and LPSN will continue to support research into prokaryotes.

## Data Availability

The provided TYGS data can be downloaded free of charge in various formats (e.g. CSV, XLS, JSON, PDF, SVG, PNG, PhyloXML, Newick, and BibTeX), provided that the source is properly cited [17, 32] when used in other works. Information obtained from LPSN is available under the CC BY-SA 4.0 license and should also be properly cited [16, 17]. Registration is necessary to access the LPSN download files and API, but this process is free of charge and simple.
